# Efficacy of Single-Dose Albendazole for the Treatment of Soil-Transmitted Helminthic Infections among School Children in Rwanda—A Prospective Cohort Study

**DOI:** 10.3390/ph16020139

**Published:** 2023-01-17

**Authors:** Joseph Kabatende, Abbie Barry, Michael Mugisha, Lazare Ntirenganya, Ulf Bergman, Emile Bienvenu, Eleni Aklillu

**Affiliations:** 1 Division of Clinical Pharmacology, Department of Laboratory Medicine, Karolinska Institutet, Karolinska University Hospital Huddinge, 14186 Stockholm, Sweden; 2 College of Medicine and Health Sciences, University of Rwanda, KK 737, Kigali P.O. Box 4285, Rwanda; 3 Rwanda Food and Drugs Authority, Nyarutarama Plaza, KG 9 Avenue, Kigali P.O. Box 1948, Rwanda

**Keywords:** efficacy, mass drug administration, albendazole, soil-transmitted helminths, school children, preventive chemotherapy, hookworms, *Trichirus trichiura*, *Ascaris lumbricoides*, Rwanda

## Abstract

Mass drug administration (MDA) of single-dose albendazole to all at-risk populations as preventive chemotherapy (deworming) is recommended by WHO to halt transmission of soil-transmitted helminth (STH) in endemic countries. We assessed the effectiveness of single-dose albendazole against STH infection in the western province of Rwanda, where STH prevalence remains high despite the implementation of preventive chemotherapy for over a decade. Two weeks before the scheduled MDA, 4998 school children (5–15 years old) were screened for STH infections (*Ascaris lumbricoides*, *Trichuris trichiura*, and hookworm), and 1526 children who tested positive for at least one type of STH parasite were enrolled and received single-dose albendazole (400 mg) through MDA. A follow-up stool exam was performed at three weeks post-treatment using Kato–Katz. Efficacy was assessed by cure rate (CR), defined as the proportion of children who became egg-free, and egg reduction rates (ERRs) at three weeks post-treatment. The CR and ERR for hookworms (CR = 96.7%, ERR = 97.4%) was above, and for *Ascaris lumbricoides* (CR = 95.1%, ERR = 94.6%) was borderline compared with the WHO efficacy threshold (CR and ERR ≥ 95%). However, the CR and ERR for *T. trichiura* (CR = 17.6% ERR = 40.3%) were below the WHO threshold for efficacy (CR and ERR ≥ 50%). Having moderate-to-heavy infection intensity and coinfection with another type of STH parasites were independent risk factors for lower CR and ERR against *Trichirus trichiura* (*p* < 0.001). Single-dose albendazole used in the MDA program is efficacious for the treatment and control for hookworms and *Ascaris lumbricoides* infections but not effective for *Trichirus trichiura*. An alternative treatment regimen is urgently needed to prevent, control, and eliminate STH as a public health problem.

## 1. Introduction

Soil-transmitted helminth (STH) infections are the most common infections of neglected tropical diseases (NTDs) worldwide, primarily affecting the poorest and most deprived communities [1]. It remains a heavy public health burden worldwide, with approximately 1.7 billion people reported to be infected [1]. STHs are endemic in the tropical and subtropical regions, with the highest burdens occurring in sub-Saharan Africa, the Americas, China, and East Asia [1]. The main STH species that infect people in Africa are *Ascaris lumbricoides*, *Trichuris trichiura*, and hookworm [2,3,4].Approximately 267 million pre-school children and more than 568 million school-age children who live in areas where these parasites are extensively transmitted required treatment and preventive measures in 2022 [4].

The burden of STH is mainly due to its chronic impact on health and quality of life rather than mortality. STH infections adversely affect child growth and development, nutritional status, and cognition [5]. To reduce the morbidity and mortality of schistosomiasis and STH, the World Health Assembly endorsed a resolution for regular treatment of high-risk groups, particularly school-age children, through mass drug administration (MDA) of anthelmintics particularly targeting the high-risk group, namely pre-school and school-aged children [6]. Preventive chemotherapy (PC) with single-dose albendazole (400 mg) or mebendazole (500 mg) is recommended as a public health intervention for all young children (12–23 months of age), pre-school (24–59 months of age) and school-age children, living in areas where the baseline prevalence of any STH infection is ≥20% [6]. Following the World Health Organization (WHO) recommendations, school-based MDA has been initiated in many endemic STH countries, including Rwanda.

PC has been successful in reducing the number of STH infections and in reducing the burden of disease (especially moderate and heavy infections) by averting an estimated 61,000 disability-adjusted life years (DALYs) from 2010 to 2015 [5]. Hence, the WHO goal for 2030 is morbidity control, defined as the reduction in moderate-to-heavy intensity infections to <2% in preschool-aged children (pre-SAC) and school-aged children (SAC) [7]. In response to this global burden, the national NTD control program was established in 2007, and the first MDA was delivered in 2008 after the initial disease mapping in Rwanda.

Previously the WHO intervention program had set a goal to eliminate STH as a public health problem (defined by WHO when the prevalence of moderate or high infection intensity is less than 1% of the at-risk population) by 2020 [6]. Despite the wide implementation of preventive chemotherapy for over a decade with high coverage, many endemic countries, including Rwanda, did not achieve the target of eliminating STH as a public health problem by 2020 [8,9,10]. The WHO recommends member states and NTD programs to monitor drug efficacy in case treatment failure is suspected or, regardless of suspected drug failure, when drugs are administered in PC-programs for at least four years [11]. To monitor anthelmintic drug efficacy, the WHO recommends measuring the reduction in the number of STH eggs excreted in stool after drug administration (egg reduction rate (ERR)) using either a single Kato–Katz thick smear or the McMaster method [11]. Currently, there is a need to closely monitor anthelmintic drug efficacy and to develop standard operating procedures, as highlighted in a WHO–World Bank meeting on Monitoring of Drug Efficacy in Large Scale Treatment Programs for Human Helminthiasis [12]. Many studies have used parasitological cure rates (CRs) to report the drug efficacy, and a few studies have reported efficacy using both parasitological cure rate and egg reduction rate (ERR) [13]. The use of both parasitological CR and ERR is important for the standardized comparison of results across studies from different regions [13].

Even though WHO recommends periodic administration of single-dose albendazole 400 mg or mebendazole 500 mg to control STH in populations at risk, including pre-school and school-age children, assessment of efficacy has not been performed in Rwanda since the start of MDA in 2008. The low cure rate of anthelmintic drugs, especially for *T. trichiura* (34%) was reported previously [14]. To date, the optimal dose for albendazole has not been determined and 400 mg is the standard dose regardless of age and/or weight [5]. Some studies have revealed the decreased efficacy of albendazole against STHs, especially in areas with high drug pressure but does not indicate causality, as this association may also be partially explained by differences in infection intensity prior to drug administration [15]. Few studies reported decreased efficacy albendazole for the treatment of STH infection [16,17], especially against *T. trichiura* [18]. However, combining anthelmintic drugs for STH indicated promising effectiveness against *T. trichiura* [19]. Studies have shown that albendazole is well-tolerated, and its related adverse events are mainly mild and self-limiting [20].

The WHO recommends co-administration of albendazole 400 mg with praziquantel as preventive chemotherapy in areas where both STH and schistosomiasis are co-endemic. In most African countries including Rwanda, both diseases are co-endemic, and hence praziquantel and albendazole are co-administered during MDA. As the current MDA control programs rely almost exclusively on few benzimidazole anthelmintics, regular surveillance and monitoring of drug efficacy are essential for early detection of parasite resistance so that mitigation strategies such as combination therapy to prolong the effectiveness of the existing anthelmintic drugs is preserved. The Rwanda Ministry of Health and NTD program recently revised the national NTD Strategic Plan to implement the new global strategy for the prevention and control of NTDs, including conducting regular assessments of drug efficacy during MDA. In addition to MDA, supplementary control measures include improved access to clean water, hygiene, and sanitation through a multisectoral approach that brings together policymakers, researchers, regulators, the national NTD program, and the community for better coordination.

Although albendazole has been used for many years for preventive chemotherapy and is well-tolerated in the treatment of STH, its co-administration with praziquantel in mass campaigns needs to be monitored for efficacy. Therefore, a safety and efficacy monitoring surveillance mechanism need to be established. The effectiveness of anthelmintic drugs administered in MDA campaigns has not been studied in Rwanda, and there is a need to prove its efficacy profile and possibilities of drug resistance. We conducted efficacy surveillance of single-dose albendazole among STH-infected school children living in high STH endemic districts of Rwanda.

## 2. Results

### 2.1. Socio-Demographic and Baseline Characteristics of Study Participants

A total of 4998 school children (5–15 years old) who were eligible for albendazole preventive chemotherapy were screened for STH infections, and 1526 who were positive for at least one type of STH parasite were enrolled in this cross-sectional albendazole efficacy surveillance study. Among the enrolled study participants, 45.8% were boys, and 73.5% were 10–15 years old. The nutritional status of enrolled children indicated that 36% were stunted, and 4.1% were wasted (had a weight below the recommended level for a given height).

The overall prevalence of hookworm infection was 4% (*n* = 61), *A. lumbricoides* was 69.7% (*n* = 1064), and *T. trichiura* infection was 92% (*n* = 1404). All hookworm-infected children had light infection intensity. Of the 1064 children infected with *A. lumbricoides*, 74.3% had light infection, 23.9% had a moderate infection, and 1.8% had heavy infection intensity. Of the 1404 children infected with *T. Trichiura* infections, 89.9% had light infection intensity, 9.8% had moderate infection intensity, and 0.4% had heavy infection intensity. The socio-demographic and baseline characteristics of participants are presented in Table 1.

### 2.2. Cure and Egg Reduction Rates in Comparison with WHO Reference Threshold

The cure and egg reduction rates for the three STH species in comparison with WHO reference threshold are presented in Table 2. The observed CR (96.7%,) and ERR (97.4%) for hookworms infection were above the WHO threshold (CR: ≥95 and ERR: ≥90). For *A. lumbricoides* infections, the observed CR (95.1%) was slightly above the WHO threshold (CR: ≥95), and ERR (94.6%) was slightly below the WHO threshold set for efficacy (ERR: ≥95). The observed CR (17.6%) and ERR (40.3%) for *T. Trichiura* infections were lower than the WHO standard threshold (CR and ERR: ≥50).

### 2.3. Cure and Egg Reduction Rate Startified by Pre-Treatment Infection Intensity and Coinfection Status

The observed cure and egg reduction rates stratified by pre-treatment infection intensity group and status of coinfection with other STH parasite species is presented in Table 3. CR decreased gradually with the intensity of infections. It was higher in children with light infections of *A. lumbricoides* (95.8%) and *T. Trichiura* (19.1%) compared with those with heavy infections of *A. lumbricoides* (89.5%) and *T. trichiura* (0.0%). For all types of STH parasites, the CR decreased by presence of coinfection with another type of STH parasite.

The ERR also gradually decreased with increased infection intensity and by presences of coinfection with two or more type of STH parasites. Both the CR and ERR for *A. lumbricoides* infection were below the WHO reference threshold (CR, ERR ≥ 95) among children coinfected with other STH parasites (CR = 94.7%, ERR = 94.5%). In contrast the CR and ERR among children with *A. lumbricoides* mono-infection was much higher (CR = 98.2%, ERR = 99.9%). On the other hand, both the CR and ERR for *T. trichiura* remained below the WHO reference (≥50) regardless of coinfection status. However, the CR was the lowest among those coinfected with other STH parasites (13.9%) than those infected with *T. trichiura* only (25.8%).

### 2.4. Association of Socio-Demographic and Baseline Characteristics with Cure Rates

The correlations of socio-demographic and baseline characteristics with cure rates of each STH parasite infections were assessed (Table 4). Both pre-treatment infection intensity and being co-infected with other STH parasites were significantly associated with cure rate of *T. trichiura* infection (*p* < 0.001). Though not significant, having moderate-to-heavy infections intensity, coinfection with other type of STH parasites, and being in an older age group appeared to associate with lower cure rate for *A. lumbricoides.* None of the variables tested for seemed to associate with cure rate of hookworm infection.

### 2.5. Predictors of Cure Rate at Three-Weeks Post-MDA

A univariate followed by a multivariate logistic regression analysis was performed to identify predictors of CR at three weeks post-treatment of albendazole against the three STH parasite infections (Table 5). On the univariate logistic regression model, pre-treatment of heavy to moderate infection intensity was significantly and negatively associated with a lower cure of *T. trichiura* infection (OR: 0.18, 95% CI: 0.08–0.43). Coinfections with other STH parasites was significantly associated with the cure of *T. trichiura* (*p* < 0.001) where the likelihood of cure was lower for participants with any coinfection compared with those with single infection (OR: 0.46, 95%:0.34–0.61) of the *T. trichiura* infections. On the multivariate logistic regression model, having coinfections and pre-treatment infection intensity remained significant independent predictors of cure for *T. trichiura* infection (*p* < 0.001). Though not significant, moderate-to-heavy infection before treatment and coinfection with other types of STH parasites were associated with a lower cure rate for *A. lumbricoides* after treatment. Sex, age group, stunting, and wasting were not significant predictors of cure at three-weeks post-treatment for hookworm, *A. lumbricoides*, and *T. trichiura* infections.

### 2.6. Risk Factors Associated with Infection Intensity

A univariate followed by multivariate negative binomial regression analysis was performed to identify factors associated with eggs count/gram of stool after three-weeks treatment (Table 6). In the multivariate negative binomial regression model, older aged (10–15 years old) and having coinfection with other STH parasites were independent significant risk factor for having higher egg count for *A. lumbricoides* after treatment. Though not significant, moderate-to-heavy infection before treatment were associated with higher eggs count/gram of stool for *A. lumbricoides* after treatment (*p* = 0.05). For *T. trichiura*, having coinfection with other STH parasites, and pre-treatment moderate-to-heavy infection intensity were independent significant risk factors for having higher eggs count/gram of stool in the multivariate model.

## 3. Discussion

The success of the global STH infection control strategy depends, among others, on the effectiveness of albendazole used in PC campaigns to treat STH infection and halt transmission in the community. Previously we conducted a cross-sectional, point-prevalence pharmaco-epidemiological study to investigate the outcome of decade-long preventive chemotherapy in reducing the burden of STH over time at the study districts. We reported albendazole 400 mg preventative chemotherapy performed for over a decade has successfully lowered the burden of hookworm infection and slightly reduced *A. lumbricoides* infection, but with no significant impact on the burden of *T. trichiura* infection in study districts [8]. The current drug-efficacy study was conducted as a follow-up to our previous study, investigating whether the high prevalence of STH in the study area (despite preventive therapy for 10 years) was due to the reduced effectiveness of albendazole in killing the parasite. In this efficacy surveillance 1526 STH-infected school children from our previous study were enrolled and received 400 mg of single-dose albendazole in MDA campaigns. Efficacy was assessed by examining the cure rates and infection intensity reduction rates after a three-week follow-up period, as recommended by the WHO [11,21].

Results of the current study indicate that albendazole is effective against hookworm, borderline effective against *A. lumbricoides*, and less effective against *T. trichiura*. Recently, we reported that 77.7% of the school children had at least one STH parasite infection at the study districts despite the implementation of biannual albendazole deworming program for >10 years [8]. This efficacy surveillance study was in alignment with WHO recommendations to assess the efficacy of anthelminthic drugs used in PC when reduced drug efficacy is observed or when PC program is implemented for more than four years [11]. The study findings revealed that albendazole used in the MDA program is efficacious for the treatment and control for hookworms and *A. lumbricoides* infections but not effective for the treatment and control of *T. Trichiura*. To our knowledge, this is the first extensive large sample size albendazole efficacy surveillance study targeting the three common STH parasites since the start of MDA implementation in 2008 in Rwanda, and in sub–Saharan Africa at large.

The observed albendazole CRs and ERR for hookworms (CR: 96.7%, ERR 97.4%) were above the WHO-recommended threshold for efficacy (Table 2). Although the observed CR for *A. lumbricoides* (CR: 95.1%, ERR: 94.6%) passed the WHO-recommended threshold for efficacy, the ERR was slightly below the WHO threshold (ERR: ≥95) [11]. Single-dose albendazole treatment resulted in a significant reduction in the infection intensity of hookworms and *A. lumbricoides* (Table 3). Older age group and coinfections with other STH parasite were significant risk factors for lower egg reduction rates for *A. lumbricoides* (Table 6). No significant effect of sex or nutritional status (stunting and wasting) on albendazole efficacy was observed. Previous studies have also reported a reduced efficacy of albendazole against *A. lumbricoides* in Rwandan school children [17] and this aligns with our current study findings that reported ERR of 94.6% in reference to the 95% WHO threshold for efficacy.

On the other hand, both the CR and ERR observed for *T. trichiura* (CR = 17.6% ERR = 40.3%) were below the WHO efficacy threshold (CR and ERR: ≥50%) (Table 2). The majority of *T. trichiura*-infected children with light infection (80.9%), moderate infection (96.6%), and all children who had heavy infection, remained uncured. Pre-treatment infection intensity and coinfections with other STH parasites were significantly associated with lower CR and ERR against *T. trichiura* infection. Our finding aligns with previous studies that reported infection intensity affects drug efficacy, and drug efficacy varies between STH species [22]. The lack of albendazole effectiveness against *T. trichiura* infection found in this study further explains why the burden of STH infections, particularly *T. trichiura* infection, remained a public health burden in the western province of Rwanda, despite biannual MDA interventions for >10 years [8]. The standard single-dose of albendazole 400 mg during preventative chemotherapy successfully reduced the burden of hookworm infection and slightly reduced *A. lumbricoides* infection but had no significant impact on *T. trichiura* infection as set by WHO threshold, this continues to pose a threat to public health [8,11]. Our finding is in line with a systematic review and meta-analysis study that reported albendazole to be effective against hookworm infection but less effective against *T. trichiura* [18]. The reduced efficacy of albendazole against *T. trichiura* was also reported previously (CR 1.1%; ERR 46.0%) and this calls for a better alternative for the treatment of *T. trichiura* [8,23,24].

Evidence from a recent clinical trial confirms that albendazole, even at higher doses, is ineffective for treating *T. trichiura* infections; and therefore, novel treatments or combination therapy should be considered to control and ultimately eliminate STH as a public health problem [25]. A recent randomized clinical trial that investigated the efficacy of 400 mg, 600 mg, and 800 mg of albendazole or placebo in school children indicated low efficacy against *T. trichiura* in school children and by all studied doses [25]. The search for alternative treatment against *T. trichiura* infection is being undertaken, including albendazole combination therapy with oxantel pamoate or with moxidectin [18,26] though so far with limited outcome. A four-arm, randomized controlled trial assessing the efficacy and safety of albendazole plus ivermectin, albendazole plus mebendazole, albendazole plus oxantel pamoate, and mebendazole alone revealed *T. trichiura* not responding to the treatment alternatives [27].

Findings from various efficacy surveillance studies underscore the need for improved or better treatment strategies to control and eliminate STH species, notably *A. lumbricoides* and *T. trichiura* in endemic regions. Although albendazole PC reduced the burden of STH, apparently interventional measures taken so far may not be adequate to control and ultimately eliminate STH as a public health problem as observed in many endemic countries, including Rwanda [8,23,24]. The Rwanda NTD program and the Ministry of Health recently revised the national NTD Strategic Plan to implement the new global strategy for the prevention and control of NTDs, including conducting regular assessments of drug efficacy during MDA [28]. A continuous albendazole efficacy monitoring study in endemic areas is evident for the early detection of drug effectiveness. This is supported by our study findings and justifies why drug effectiveness assessment studies are regularly needed for better program implementation and treatment outcome measurements.

In deworming programs, albendazole is provided to treat and control the burden of the three common STH parasites in children. Our result indicates the effectiveness of albendazole against hookworms and *A. lumbricoides* infection but not *T. trichiura* infections. As we reported previously, the repeated use of albendazole MDA over the years reduced the burden of hookworms and *A. lumbricoides* infections in the study districts [8]. Therefore, while the search for a better drug against *T. trichiura* continues, we recommend the national NTD program continue with albendazole MDA along with other supplementary preventive measures such as improvements in water, sanitation, and hygiene (WASH) to avoid contamination and re-infections as part of the strategy to eliminate STH as a public health problem in Rwanda.

## 4. Materials and Methods

### 4.1. Study Area, Population, and Participants

This cross-sectional efficacy surveillance of albendazole was conducted during the MDA campaign in four districts of the western province of Rwanda in April 2019. The three districts located around the belt of lake Kivu namely, Rubavu, Nyamasheke, and Rusizi, were selected for this study based on epidemiological data related to high endemicity of STH. Within each district, two schools were selected based on previous STH prevalence data, and the number of school-age children attending. A sample proportion of each school to contribute to the whole study sample was based on student population size. School children were systematically sampled in each class using class lists.

The study participants were school-age children who were infected with at least one STH parasite (hookworm, *Ascaris lumbricoides*, and *Trichuris trichiura*). A total of 4998 school-age children (5–15 years old) were screened for *A. lumbricoides*, *T. trichiura,* and hookworm. A total of 1526 school children who tested positive for at least one STH parasite were enrolled in this efficacy surveillance study and were eligible for albendazole preventive chemotherapy as per the Rwandan national NTD public health program.

### 4.2. Ethics

Ethical approval to conduct the study was obtained from the Rwandan National Ethics committee and National Health Research Committee of the Ministry of Health, Rwanda. School children whose parents/guardians provided written informed consent were included in the study. Children whose parents or guardians were not willing to provide informed consent and or dissent were not included in the study.

### 4.3. Drug Administration and Follow-Up

Study participants received albendazole 400mg as part of the MDA campaign following the national and WHO MDA guideline [29]. MDA was administered to all children attending the study schools regardless of their STH infection status as scheduled by the Rwanda Ministry of Health [17]. The study team had no role in the MDA planning or administering the drug. Children participating in the study were provided a light snack before albendazole administration. Three weeks after albendazole MDA, the enrolled school-age children followed-up for efficacy outcome measurement.

### 4.4. Screening for STH Parasite Species

Fresh stool samples were collected from study participants two weeks before, and three weeks after albendazole MDA for STH screening and efficacy follow-up, respectively. Two Kato–-Katz smears were prepared from the collected stool sample using a template of 41.7 mg and processed as recommended by WHO [30]. Duplicate slides were prepared from each stool sample and read independently by the two laboratory technicians. Lab technicians from the National Reference Laboratory, Hospitals, and Health Centers analyzed samples, and senior lab technicians conducted quality control and analyzed up to 10% of all stool samples examined per day. Before data collection, all lab technicians were trained and supervised by research coordinators, and children were educated on how to provide a stool sample of their own, avoiding contamination with urine.

The intensity of infection for each STH parasite was categorized as “light”, “moderate” or “heavy” based on fecal egg counts per gram of stool (epg) using the cut-off threshold set by the WHO [30] as *T. trichiura*; light (1–999 epg), moderate (1000–9999 epg), heavy (≥10,000 epg), *A. lumbricoides*; light (1–4999 epg), moderate (5000–49,999 epg), heavy (≥50,000 epg) and hookworm; light (1–1999 epg), moderate (2000–3999 epg), heavy (≥4000 epg).

### 4.5. Data Processing and Statistical Analysis

A complete participant form with a unique identification number and stool sample was handed to the laboratory technician to screen and complete laboratory exam results. Data were collected in the electronic database and imported in STATA 13 for processing and analysis. Outcome variables were categorized as positive and negative for individual STH parasites (hookworm, *A. lumbricoides*, and *T. trichiura*). A participant was STH-positive if he or she had at least one egg count of that species on one of two Kato–Katz slides tested. Associations between cure rates and independent categorical variables were analyzed using the Chi-square test and Fisher’s exact test. Predictors of the cure rate for each STH species were first analyzed using univariate followed by multivariate logistic regression analysis. Factors associated with post-treatment egg count/gram of stool were analyzed using a negative binomial regression model. Predictor variables with *p* ≤ 0.2 in the univariate analyses were entered into the multivariate model for analysis. A *p*-value < 0.05 was considered statistically significant. The study outcome was efficacy as measured by CR and ERR based on the thick smear Kato–Katz method at three weeks post-MDA treatment. The CR was defined as the proportion of egg-positive children before treatment who became egg free at three weeks post-MDA. The ERR was calculated as 100 times (1 − (Arithmetic mean of epg after treatment/Arithmetic mean of epg before treatment)), as recommended by the WHO [11].

## 5. Conclusions

Our finding indicates that single-dose 400 mg albendazole used in deworming program to control STH is effective against hookworm, borderline effective against *A. lumbricoides*, and not effective against *T. trichiura* as per the WHO threshold set for efficacy. Pre-treatment moderate-to-heavy infection intensity and concurrent coinfections with two or more STH parasites are independent risk factors for lower albendazole cure rates and egg reduction rates for *A. lumbricoides* and *T. trichiura* infections. The current single-dose albendazole-based PC intervention strategy may not effectively eliminate STH, particularly *T. trichiura,* as a public health problem in accordance with the global targets and milestones to prevent, control, and eliminate the disease by 2030. The study highlights the need for better alternative regimens to treat *T. trichiura* infection. As anthelmintic drugs used in MDA programs are provided to all at-risk populations without prior diagnosis or follow-up, regular efficacy surveillance is recommended for early detection and management of drug resistance, especially in endemic regions with a high burden of STH infections. Complementary strategies including safe water, good and appropriate nutrition, and multisectoral interventional strategies coordination may contribute to the elimination of the disease.

## Figures and Tables

**Table 1:** Socio-demographic and baseline characteristics of study participants.

Variable			*N*	%
Sex		Male	699	45.8
		Female	827	54.2
Age categories		5–9 years	405	26.5
		10–15 years	1121	73.5
District		Rubavu	682	44.7
		Nyamasheke	311	20.4
		Rusizi	533	34.9
School		Rambo	317	20.8
		Rubona	365	23.9
		Buhokoro	171	11.2
		Mukoma	140	9.2
		Bugumira	183	12
		Nkombo	350	22.9
Consistency of stool		Formed	29	1.9
		Soft	1492	97.8
		Loose	2	
		Watery	3	0.2
Stunting status (HAZ)		Non-stunted	976	64
		Stunted	550	36
Wasting status (BAZ)		Not wasted	1463	95.9
		wasted	63	4.1
Hookworms		Light intensity	61	100
*Ascaris lumbricoides*		Light intensity	791	74.3
		Moderate intensity	254	23.9
		Heavy intensity	19	1.8
*Trichirus trichiura*		Light intensity	1262	89.9
		Moderate intensity	137	9.8
		Heavy intensity	5	0.4
Coinfections with other STH parasite species	Hookworms	No coinfection	4	6.6
		Coinfection with AL or TT	57	93.4
	*Ascaris lumbricoides*	No coinfection	114	10.7
		Coinfection with HW or TT	950	89.3
	*Trichirus trichiura*	No coinfection	438	31.2
		Coinfection with HW or AL	966	68.8

HAZ: height-for-age z-scores; BAZ: BMI-for-age z-scores; HW: Hookworms; AL: Ascaris lumbricoides; TT: Trichirus trichiura.

**Table 2:** Cure and egg reduction rates for the STH species in comparison with the WHO threshold recommended for efficacy [11].

Type of STH Infection	Cure Rate (CR)				Egg Reduction Rate (ERR)		
	n	Number Cured	CR, % (95% CI)	WHO Threshold for CR	Mean (SD)	ERR, %	WHO Threshold for ERR
Hookworms	61	59	96.7 (92.2–101.2)	≥95	1.8 (11.1)	97.4	≥90
*Ascaris Lumbricoides*	1064	1012	95.1 (93.8–96.4)	≥95	288.8 (3065.5)	94.6	≥95
*Trichirus trichiura*	1404	247	17.6 (15.6–19.6)	≥50	301.2 (866.9)	40.3	≥50

STH: Soil-transmitted helminth; CI: confidence interval.

**Table 3:** Cure rate and egg reduction rate stratified by pre-treatment infection intensity and by status of coinfection with other type of STH parasites.

STH Parasites		*N*	Number Cured	Cure Rate, % (95% CI)	WHO Reference for CR	Mean (SD)	ERR, %	WHO Reference for ERR
**By pre-treatment infection intensity**								
Hookworms	Light	61	59	96.7 (96.7–101.2)	≥95	1.77 (11.1)	97.5	≥90
*Ascaris lumbricoides*	Light	791	758	95.8 (94.4–97.2)	≥95	790.68 (5738.7)	95.5	≥95
	Moderate or heavy	273	254	93.0 (90.0–96.1)		115.57 (1090.4)	89.6	
*Trichirus trichiura*	Light	1262	241	19.1 (16.9–21.3)	≥50	1047.3 (1977.3)	62.3	≥50
	Moderate or heavy	142	6	4.2 (0.9–7.5)		217.3 (573.8)	12.4	
**By coinfection status**								
Hookworms	No coinfection	4	4	100	≥95	0 (0)	100	≥90
	Coinfection with AL or TT	57	55	96.5 (91.7–101.2)		1.89 (11.5)	97.4	
*Ascaris lumbricoides*	No coinfection	114	112	98.2 (95.8–100.6)	≥95	1.47 (13.7)	99.9	≥95
	Coinfection with HW or TT	950	900	94.7 (93.3–96.2)		323.3 (3242.7)	94.5	
*Trichirus trichiura*	No coinfection	438	113	25.8 (21.7–29.9)	≥50	164.4 (490.6)	36.5	≥50
	Coinfection with HW or AL	966	134	13.9 (11.7–16.1)		363.2 (985.5)	41	

STH: soil-transmitted helminth; CI: confidence interval; HW: hookworms; AL: *Ascaris lumbricoides*; TT: *Trichirus trichiura*.

**Table 4:** Factors associated with cure rates of albendazole against hookworm *A. lumbricoides* and *T. Trichiura* infections.

Variable		Hookworms (*N* = 61)			*Ascaris lumbricoides* (*N* = 1064)				*Trichirus trichiura* (*N* = 1404)			
		*N*	Cure Rate, % (*n*)	*p*	*N*	Cure Rate, % (*n*)	X^2^	*p*	*N*	Cure Rate, % (*n*)	X^2^	*p*
Sex	Male	27	96.3% (26/27)	0.69	493	95.3% (470/493)	0.09	0.76	647	17% (110/647)	0.29	0.59
	Female	34	97.1% (33/34)		571	94.9% (542/571)			757	18.1% (137/757)		
Age categories	5–9 years	13	100% (13/13)	0.61	286	97.2% (278/286)	3.68	0.06	370	20% (74/370)	2.0	0.16
	10–15 years	48	95.8% (46/48)		778	94.3% (734/778)			1034	16.7% (173/1034)		
Stunting status (HAZ)	Normal	34	94.1% (32/34)	0.31	681	95.6% (651/681)	0.95	0.33	893	17.9% (160/893)	0.18	0.67
	Stunted	27	100% (27/27)		383	94.3% (361/383)			511	17.0% (87/511)		
Wasting status (BAZ)	Normal	56	98.2% (55/56)	0.16	1019	95.2% (970/1019)	0.32	0.57	1346	17.6% (237/1346)	0.005	0.94
	wasted	5	80% (4/5)		45	93.3% (42/45)			58	17.2% (10/58)		
Pre-treatment Infection intensity	Light	61	96.7% (59/61)		791	95.8 (758/791)	3.39	0.07	1262	19.1% (241/1262)	19.5	<0.001
	Moderate or heavy	0			273	93.0% (254/273)			142	4.2% (6/142)		
Coinfections with other STH parasite species	Mono-infection	4	100% (4/4)	0.12	114	98.2% (112/114)	2.26	0.10	438	25.8% (113/438)	29.6	<0.001
	Dual or triple coinfection	57	96.5% (55/57)		950	94.7% (900/950)			966	13.9% (134/966)		

HAZ: height-for-age z score; BAZ: BMI-for-age z-scores; STH: soil-transmitted helminth.

**Table 5:** Predictors of cure rate at three weeks post-single-dose albendazole mass drug administration.

Variables		Hookworm			*A. lumbricodes*					*T. trichiura*				
		Cured N (%)	cOR (95% CI)	*p*	Cured N (%)	cOR (95% CI)	*p*	aOR (95% CI)	*p*	Cured N (%)	cOR (95% CI)	*p*	aOR (95% CI)	*p*
Sex	Female	33 (97.1)	1	0.87	542 (94.9)	1	0.76			137 (18.1)	1	0.59		
	Male	26 (96.3)	0.78 (0.04–14.3)		470 (95.3)	1.09 (0.62–1.91)				110 (17)	0.93 (0.70–0.22)			
Age categories	5–9 years	13 (100)	1		278 (97.2)	1	0.06	1	0.06	74 (20)	1	0.16	1	0.16
	10–15 years	46 (95.8)	Omitted		734 (94.3)	0.48 (0.22–1.03)		0.48 (0.22–1.04)		173 (16.7)	0.80 (0.59–0.09)		0.80 (0.59–1.09)	
Stunting (HAZ)	Non-stunted	32 (94.1)	1		651 (95.6)	1	0.33			160 (17.9)	1	0.67		
	Stunted	27 (100)	Omitted		361 (94.3)	0.94 (0.71–1.25)				87 (17)	0.94 (0.71–1.25)			
Wasting (BAZ)	Non-wasted	55 (98.2)	1	0.09	970 (95.2)	1	0.57			237 (17.6)	1	0.94		
	Wasted	4(80)	0.73 (0.03–1.52)		42 (93.3)	0.97 (0.49–1.96)				10 (17.2)	0.97 (0.49–0.96)			
Infection Intensity	Light	61 (100)	-		758 (95.8)	1	0.07	1	0.12	241 (19.1)	1	<0.001	1	**<0.001**
	Moderate-to-heavy	0 (0)	-		254 (93.04)	0.58 (0.32–1.04)		0.62 (0.34–1.13)		6(4.2)	0.18 (0.08–0.43)		0.21 (0.09–0.5)	
* Coinfections with other STH parasite species	Mono-infection	4 (100)	1		112 (98.3)	1	0.12	1	0.16	113 (25.8)	1	<0.001	1	**<0.001**
	Dual or triple coinfection	55 (96.5)	Omitted		900 (94.7)	0.32 (0.77–1.34)		0.36 (0.09–1.53)		134 (13.9)	0.46 (0.34–0.61)		0.5 (0.38–0.67)	

* Coinfections with other STH parasites; cOR: crude odds ratio; aOR: adjusted odds ratio; CI: confidence interval; HAZ: height-for-age z score; BAZ: BMI-for-age z-scores; STH: Soil-transmitted helminth.

**Table 6:** Negative binomial regression model for factors associated with eggs count/gram of stool after treatment.

Variables		*Ascaris lumbricoides*						*Trichirus trichiura*					
		Univariate Analysis			Multivariate Analysis			Univariate Analysis			Multivariate Analysis		
		β (S.E)	95% CI	*p*	β (S.E)	95% CI	*p*	β (S.E)	95% CI	*p*	β (S.E)	95% CI	*p*-Value
Age categories	5–9 years	1		0.005	1	0.01–0.69	0.02	1					
	10–15 years	16.47 (16.47)	2.32–116.95		13.15 (14.75)	1.45–18.55		1.09 (0.12)	0.88–1.35	0.75			
Stunting	Non-stunted	1		0.93				1					
	Stunted	0.92 (0.86)	0.14–5.85					0.89 (0.09)	0.73–1.08	0.25			
Wasted	Non-wasted	1		0.75				1					
	Wasted	0.44 (0.99)	0.005–36.54					0.91 (0.22)	0.56–1.46	0.68			
Infection intensity	Light	1		0.059	1	0.02–1.03	0.05	1			1		<0.001
	Moderate-to-heavy	6.84 (6.96)	0.93–50.24		6.69 (6.59)	0.97–46.10		4.82 (0.74)	3.57–6.51	<0.001	4.46 (0.68)	3.30–6.01	
* Coinfections	Mono-infection	1		<0.001	1		0.015	1			1		<0.001
	Dual or triple coinfection	219.36 (314.84)	13.17–3654.88		54.07 (88.48)	2.19–1335.91		2.21 (0.23)	1.80–2.70	<0.001	1.87 (0.18)	1.54–2.27	

* Coinfections with other STH parasites; CI: confidence interval.

## Data Availability

Data are contained within the article.
